# Book Notices

**Published:** 1899-05

**Authors:** 


### BOOK NOTICES.

The International Medical Annual, 1899. A work of reference for medical practitioners. Alphabetically arranged. Combines the features of an annual retrospect with those of a medical encyclopedia. Copiously illustrated with elegant plates, in colors and black and white. Each volume contains enirely new matter. Seventeenth year. 8vo, cloth, about 700 pages. Price, $3.00 net, post or express paid. E. B. Treat & Co., Publishers, 241-243 West Twenty-third Street, New York.

It will be seen that this work is something more than a mere retrospect of the past year. It includes a series of articles intended to bring the reader's knowledge up to date on subjects of modern investigation, and the present volume contains new matter of practical interest concerning almost every known disorder.

Among the special articles will be found the following: "Practical X-Ray Work," by R. Norris Wolfenden, M. D., B. A.; "Advances in Skull Surgery," by Seneca D. Powell, M. D.; "Surgical Treatment of Paralysis," by Drs. Robert Jones, F. R. C. S., and A. H. Tubby, M. S., M. B. These articles will be freely illustrated, chiefly by reproductions from photographs. An excellent article on "Climatic Treatment of Consumption," by F. de Havilland Hall, M. D., F. R. C. P., as well as one on "Legal Decisions Affecting Medical Men," by William A. Purrington, A. B., LL. M., will be found interesting and pertinent. There is also an article on "The Chief Pathogenic Bacteria in the Human Subject," with descriptions of their morphology and methods of microscopical examination by S. G. Shattock, F. R. C. S., the Pathological Curator of the Museum of the Royal College of Surgeons, London, illustrated by a series of finely colored plates.

The *Annual* is now a standard work of reference in all parts of the world, and the publishers feel some pride in asserting that no medical work of such a widely international character has been previously issued by the medical press in any country, which offers so much at so small a price.

The Antikamnia Chemical Company has just issued a handsome Fœtal Chart and Parturition Calendar.

It consists of three cards giving six pages of the progress of gestation for each month, finely illustrated by colored diagrams, so that the obstetrician, by consulting it, may form an accurate idea at a glance of what is the development of the fœtus at any given period of its inter-utero life. It is issued solely to the members of the medical profession, and it is desired that every practitioner shall have a copy of it. Those who fail to receive it may procure it by writing to the company. No attention will be paid to such requests, however, unless they are accompanied with the professional card or other evidence that the writer is a practicing physician. Address The Antikamnia Chemical Company, 1723 Olive Street, St. Louis, Mo.

